# Prenatal diagnosis of an acardiac twin

**DOI:** 10.1590/0100-3984.2016.0133

**Published:** 2018

**Authors:** Jamylle Geraldo, Cesar Rodrigo Trippia, Maria Fernanda F. S. Caboclo, Raphael Rodrigues de Lima, Gabriel Cleve Nicolodi

**Affiliations:** 1 Hospital São Vicente - Funef, Curitiba, PR, Brazil

**Figure 1:**
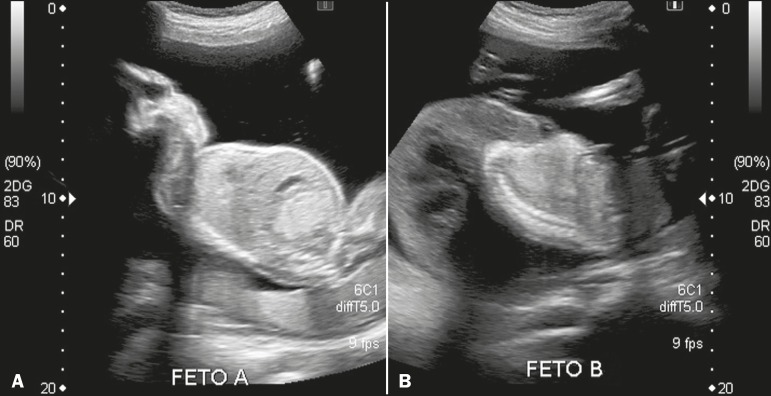
Ultrasound showing comparative images, in the sagittal plane, of a morphologically normal fetus (A) and of a fetus with a bizarre anatomical configuration (B), including the absence of brain formation, no upper or lower limb buds, and hydrops fetalis.

Dear Editor,

A 32-year-old female patient who was pregnant with twins presented for a regular prenatal checkup with her obstetrician at 25 weeks of gestation. It was her second pregnancy, and she had carried the first pregnancy to delivery. She was asymptomatic. Ultrasound showed that one twin was morphologically normal and that the other was hydropic, with involution of the brain and only the most rudimentary cardiac tissue (Figure 1).

**Figure 2:**
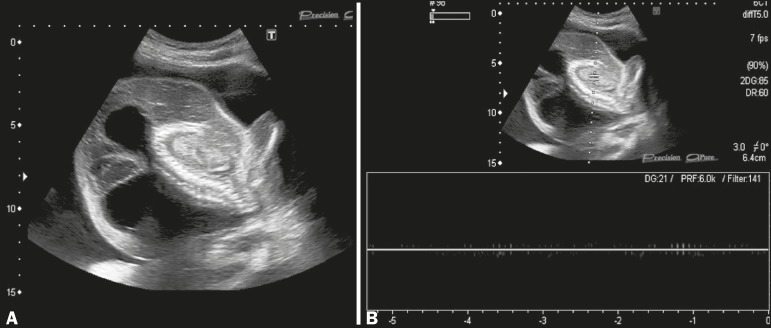
Ultrasound images, in the sagittal plane, showing structural disorganization of the abnormal fetus (A), with absence of the cephalic pole, no upper or lower limb buds, only rudimentary cardiac tissue, and hydrops fetalis. Spectral Doppler analysis detected no fetal cardiac activity (B).

Recent studies have highlighted the importance of imaging examinations in fetal medicine^(1-3)^. Multiple pregnancies are subject to various complications, the rarest of which is an acardiac fetus, a complication seen in only 1% of all monochorionic twin pregnancies^(4)^. Although the pathophysiology of an acardiac twin is not well known, it is believed that there are vascular anastomoses that divert blood from the morphologically normal twin to the acardiac twin, a condition known as twin reversed arterial perfusion. The acardiac twin almost always presents involution of the brain, together with the absence or malformation of other organs (Figure 2). The normal twin can suffer complications such as heart failure, polyhydramnios, hydrops fetalis, and growth restriction, as well as being at high risk for fetal death^(4-6)^.

Approximately 20% of acardiac fetuses have vestiges of cardiac tissue or a rudimentary heart. Therefore, it would be correct to call them pseudoacardiac fetuses. That makes the case reported here even more rare, because it involves a pseudoacardiac twin^(4,6)^.

The morphological diagnosis of an acardiac twin is made by fetal ultrasound and is based on the following criteria^(6)^: monochorionic twin pregnancy; reverse flow in the umbilical cord and descending aorta; presence of arterio-arterial anastomoses; and partial or complete absence of the heart in one of the fetuses. An acardiac twin can sometimes be confused with a teratoma. The two can be differentiated by identifying the umbilical cord and some degree of organization of the body of the acardiac fetus^(6)^.

In 50-75% of cases of an acardiac twin, the use of the watchful waiting strategy is associated with the death of the structurally normal twin, due to heart failure and hydrops fetalis. In the case presented here, the pregnancy was monitored to term through the use of serial examinations, and there were no complications for the structurally normal fetus or for the mother. The treatment, when necessary, is still controversial. It involves blocking the blood flow to the acardiac twin if the structurally normal twin shows some impairment. The main surgical techniques are aimed at occlusion of the umbilical cord-by ligation with a suture, clamping with bipolar forceps, photocoagulation, or ligature/section of the cord—or obliteration of the circulation with absolute alcohol. The survival rate for the structurally normal fetus can be as high as 75% when some intervention is implemented^(6,7)^.

An acardiac fetus is a rare complication of multiple monochorionic pregnancies and can be diagnosed through the use of a widely accessible method. Early identification of an acardiac twin can avert a fatal outcome for the structurally normal twin.
